# Post‐operative discomforts in children after extraction of primary teeth

**DOI:** 10.1002/cre2.316

**Published:** 2020-08-23

**Authors:** Claire Baillargeau, Serena Lopez‐Cazaux, Hugo Charles, Aline Ordureau, Sylvie Dajean‐Trutaud, Tony Prud'homme, Isabelle Hyon, Assem Soueidan, Brigitte Alliot‐Licht, Emmanuelle Renard

**Affiliations:** ^1^ Centre Hospitalier Universitaire de Nantes Pole Hospitalo‐Universitaire 4 Nantes France; ^2^ Université de Nantes UFR d'Odontologie Nantes France; ^3^ Centre Hospitalier Universitaire de Nantes Direction de la recherche, Service de méthodologie Nantes France

**Keywords:** acetaminophen, analgesic, pain, paracetamol, primary tooth, tooth avulsion

## Abstract

**Objectives:**

This prospective observational study aimed to evaluate discomfort after extraction of deciduous teeth under local anesthesia. The primary objective was to describe the prevalence of post‐extraction pain (PEP), post‐extraction bleeding (PEB), post‐extraction biting injury (PEBI), and analgesic usage in children. The secondary objective was to define whether it is possible to determine a profile of patients or a type of extraction procedure predictive to PEP, administration of analgesics, PEB, or PEBI.

**Methods:**

One hundred and twenty‐five children, aged 3–13 years, with indications of at least one deciduous tooth extraction, were included. Immediately after extraction, information concerning the patient and the extraction were collected. Eighteen to 32 hr after extraction, parents were called by phone to request reports concerning the onset and intensity of PEP assessed using the Wong‐Baker Faces (WBF) scale, the administration of paracetamol (acetaminophen) to their children, and the appearance of PEB and/or PEBI.

**Results:**

Of the children, 37.3% reported PEP (WBF ≥2), but 23.3% of these children did not receive any analgesic drugs to help relieve pain. Pain appeared before 3 hr after extraction in 69% of the children. Higher incidences of PEP and usage of analgesics were found both in the group of children with unfavorable socioeconomic level compared to favorable level and in the group with pre‐operative pain compared to no pre‐operative pain (*p* < .05).

**Conclusions:**

About a third of the children reported pain after extraction, but the instructions for pain relief were not followed by all parents. The socioeconomic level of the young patient and the pain felt during the extraction were important predictors of discomfort. Therefore, our study could help the dentist to provide information on predicted post‐operative discomfort and to allow suitable care depending on the patient's profile or procedure.

## 1 INTRODUCTION

**WHY THIS PAPER IS IMPORTANT FOR PEDIATRIC DENTISTS?**

- The results of this study can be used by clinicians after extraction of deciduous teeth.
- Given the costs and possible problems related to unjustified use of painkillers or the absence of analgesic with pain, taken together our data could lead to more appropriate prescribing decisions to preventatively treat the appearance of post‐operative pain.

Prevention and management of post‐extraction pain (PEP), post‐extraction lip or cheek biting injury (PEBI), and/or post‐extraction bleeding (PEB) following deciduous tooth removal are integral parts of treatment in pediatric dentistry. In fact, neglecting experiences perceived as unpleasant by the child, such as local anesthetic administration, tooth extraction, and post‐operative discomfort, can lead to the development of anxiety and interfere with the acceptance of dental treatment during future visits (Pala, Nuvvula, & Kamatham, 2016). Conversely, informing young patients and their parents about expected post‐extraction problems and prescribing medications to manage pain should increase children and parents' confidence in their dentists. However, except for orthodontic treatment and third molar extraction (Weil et al., 2007), no guidelines exist for the use of analgesics in children undergoing dental treatment without general anesthesia (Berlin et al., 2019).

Paracetamol (acetaminophen) is a common analgesic with high usage and availability (Radman et al., 2019). However, there is a risk of toxicity from overdose, and hypersensitivity reactions to paracetamol appear to be increasing (Rutkowski, Nasser, & Ewan, 2012). A recent systematic literature review conducted to assess the adverse event profile of paracetamol in the general adult population demonstrated a consistent dose–response relationship between paracetamol at standard analgesic doses and adverse events (Roberts et al., 2016). These authors suggested a considerable degree of toxicity, especially at the upper end of standard analgesic doses in the treatment of osteoarthritis joint pain and low back pain. In addition, even if the evidence is inconclusive, the association between paracetamol and asthma is under debate (Berlin et al., 2019). Although several large observational studies confirm better side effect profiles for paracetamol compared with traditional non‐steroidal anti‐inflammatory drugs, every prescribing decision should involve a calculation of risk versus benefit, and when benefit is uncertain, more careful consideration of paracetamol usage is required (Berlin et al., 2019; Deshpande, Bhargava, & Gupta, 2014). Moreover, a recent systematic review of paracetamol in treating all types of pain in children reached the conclusion that even if paracetamol is recommended in most guidelines, high quality clinical trials are needed to generate better evidence regarding the efficacy of this analgesic for treating pain (Radman et al., 2019).

In the literature, previous studies reported frequencies of PEP in children ranging from 38 to 42.8% (Acs, Moore, Needleman, & Shusterman, 1986; Acs, Moore, Shusterman, & Needleman, 1988; Ashkenazi, Blumer, & Eli, 2007). However, in these studies, the failure to use scale induced a risk of bias, and there was a lack of precision as to the types of extracted teeth (deciduous or permanent; Tomlinson, von Baeyer, Stinson, & Sung, 2010). Moreover, these studies are old, and anesthesia molecules, spin, as well as non‐pharmacological behavioral techniques used in contemporary pediatric dentistry should modify the data concerning the pain felt by children. In addition, in 2012 and 2016, Ashley et al. reviewed the available evidence regarding the use of pre‐operative analgesics for additional pain relief in children undergoing dental treatments (i.e., restorative, extraction, or orthodontic treatment). Nevertheless, each study was assessed as being at risk of bias, and the data of this meta‐analysis allow the authors to conclude that further research on post‐operative pain after deciduous tooth removal is warranted and will help inform the development of prescribing guidelines where appropriate (Ashley, Parekh, Moles, Anand, & Behbehani, 2012; Ashley, Parekh, Moles, Anand, & MacDonald, 2016).

Finally, to our knowledge, the prevalence of lip and cheek biting injury has not been specifically evaluated after primary tooth extraction. Some studies recall this problem after local dental anesthesia (Ashkenazi et al., 2007), but we hypothesis that in addition to the anesthesia effect, tooth removal could have an impact on the number of children who bite themselves after extraction. In addition, the incidence of PEB should be specifically explored.

The principal objective of this observational trial was to estimate the prevalence and severity of pain after extraction of deciduous teeth, the analgesic usage and reported efficacy of this analgesic in children, as well as the incidence of post‐operative lip or cheek biting and/or bleeding. The second objective was to evaluate the frequency of PEP, PEBI, and PEB, as well as the use of analgesic agents in children with regard to factors based on the patient or on the tooth and the surgery's characteristics.

## 2 METHODS

This prospective study adheres to the STROBE statement (Vandenbroucke et al., 2007) (Appendix S1) and was performed in Nantes Dental University and Hospital, France, during the period April 2016–April 2017. Clinical trials registration number: 0387817.

### 2.1 Sample

A total of 125 children, 3–13 years of age (mean 7.8 ± 2.3 years, 56.7% boys), indicated for tooth extractions for reason of tooth decay, orthodontic treatment, obstacle, infection, or traumatism, were included in this study. The exclusion criteria were patient under analgesic the day of the appointment or taking non‐steroidal anti‐inflammatory agents for 8 days before the extraction; contraindication to paracetamol/acetaminophen; incomplete mental health; extractions of permanent teeth; and/or extraction under sedation (including nitrous oxide/oxygen) and general anesthesia. Children were also excluded from the study if the parents could not be reached by phone within 32 hr after extraction or if the parents did not speak French.

Subjects' rights have been protected by the local ethic committee of Nantes (accredited by the Institutional Clinical Research and Innovation Direction), which approved this study (number RC 16‐0183 University Hospital Centre). All parents signed informed consent forms, and each child consented verbally according to their age.

### 2.2 Intervention

After application of xylocaine gel for topical anesthesia of oral mucosa, anesthesia by local infiltration of 4% articaine with adrenaline (epinephrine) (1:200,000) was administered for all the recruited children. Extraction of the deciduous tooth was then performed consistently by all the operators (undergraduate students supervised by pediatric specialized dentists) following standard protocol with routine behavioral guidance techniques. Immediately after extraction, parents were instructed with a written guide for home care of their children; the pain relief instruction was to give one adequate dose of pediatric paracetamol (based on the child's weight, using 15 mg/kg/dose), and, if necessary, others doses every 4–6 hr, not to exceed four doses in 24 hr, only when children felt pain with a score greater than or equal to 2 on the Wong Baker Face (WBF) scale. On this scale, which contains six faces, the children were asked to point to the face that displayed the amount of pain they were experiencing (WBF; 0: no pain, 2: mild pain, 4: moderate pain, and 6–10: severe to worst pain). To prevent injury to the mouth while the area remained anesthetized, the recommendation was to supervise the children and to cook mixed foods. To protect the clot that was formed and to prevent bleeding, children should not suck on the extraction site and should avoid excessive exercise for several hours. Written recommendations in the case of appearance of these discomforts were given.

Finally, parents were asked to fill out a form containing the precise time and WBF score of PEP, PEBI, and PEB, and the administration (dose and time) of analgesic drugs. Eighteen to 32 hr after extraction, a clinical research technician unaware of the extraction performed telephoned to the parents to request reports concerning PEP, administration of analgesic, PEBI and/or PEB.

A structured form was designed to obtain information on the patients' ages, sexes, and socioeconomic levels differentiated by their health insurance status (unfavorable status receiving state medical aid or complementary health care), as well as cooperation of the child before and during anesthesia or extraction, influence of the accompanying person, number of previous extractions, and dental hygiene. Moreover, details about the type of extraction, the indication of extraction (tooth decay, orthodontic treatment, obstacle, infection, or traumatism), the type of tooth (incisor‐canine or molar), the level of root resorption, number of doses of local anesthetic, and presence of pre‐existing or pre‐operative pain were reported by the operator on a faces pain scale, revised (FPS‐R).

The data were collected to ascertain the number and relative proportion of children who felt pain. Then, data were analyzed to determine the possible association between the type of patient or the nature of intervention and the report of discomfort.

### 2.3 Sample size calculation

The prevalence of post‐operative pain after extraction of primary teeth is reported to be around 40% in the literature (Acs et al., 1986, 1988; Ashkenazi et al., 2007), but after discussions with pediatric dentists, this expectation of post‐operative pain seems to be higher than what is observed. Given this probability, in order to have enough patients for the second objective, a sample size of 100 was selected for the calculation, with the expectation that from 20 to 40 patients would experience discomfort. To anticipate that 25% of the parents would not fill out the survey forms, the calculated sample size of 100 was increased to 125 patients.

### 2.4 Statistical analysis

Incidence of PEP was estimated with a 95% confidence interval. Univariate logistic regression models were used to analyze factors associated to post‐operative pain, analgesic use, and post‐operative bleeding. Results were presented with OR and their 95% confidence interval. A *p*‐value less than .05 was considered statistically significant. Statistical analyses were realized with SAS Software version 9.4.

## 3 RESULTS

A total of 125 children were initially included. Five were excluded because they could not be reached by telephone. All patients who could be reached by telephone agreed to respond to the questionnaire (100% response rate).

### 3.1 Incidence of PEP, PEBI, and PEB

**FIGURE 1:**
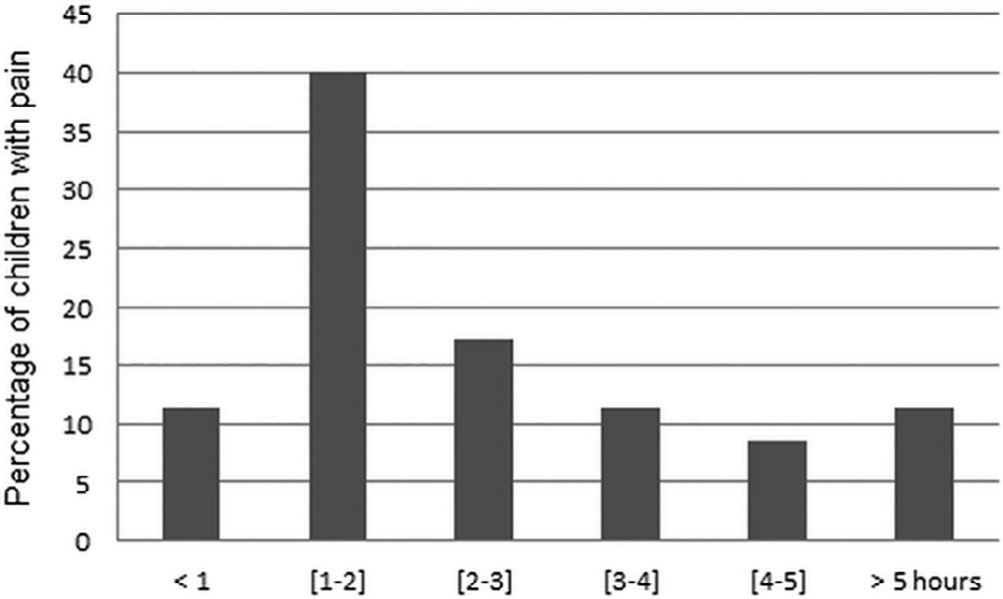
Cumulative percentage of children with pain at different time points

The incidence of pain (WBF ≥ 2) following deciduous tooth extraction was 37.3%. Among the 44 patients who had being suffering, the proportion of patients reporting mild or moderate pain reached 60.4% (39.5% WBF = 2 and 20.9 WBF = 4) (Table 1). For half the patients, pain appeared during the 2 hr after the dental extraction (Figure 1). Concerning analgesic usage, 48 of the 118 children (40.7%, two missing data) received at least one dose of paracetamol administered by their parents, but 12 children without pain (16.7% of children with WBF = 0) took an analgesic, and 10 children who reported pain (23.3% of children with WBF≥6) did not receive drugs to relieve their pain. In detail, among this group of 43 patients who experienced pain, three of the nine patients suffering moderate pain (33.3% of children with WBF = 4) and one of the 10 patients with severe or worst pain imaginable (5.9% of patients with pain WBF ≥ 6) did not receive any painkillers (Table 1). In summary, 22 of the 118 parents (18.6%, two missing data) did not follow the dentist's recommendations (Table 1). Finally, we observed that the children's pain relief was completely effective and did not require a second dose of analgesic for 88.5% of patients with WBF < 6 and for 76.5% of patients with WBF ≥ 6. Only seven (three with moderate pain and four with severe pain) of the 120 patients enrolled in our study required paracetamol a second time (Table 1), and none of the children received a third dose.

**TABLE 1:** Prevalence of post‐extraction pain, analgesic usage, post‐extraction biting injury and post‐extraction bleeding

Post‐extraction pain (PEP) reported	Frequency	Percent
No (WBF = 0)	74/118	62.7
Yes (WBF ≥ 2)	44/118	37.3
Frequency missing = 2		

PEP reported according to WBF	Frequency	Percent
WBF = 2	17/43	39.5
WBF = 4	9/43	20.9
WBF = 6	9/43	20.9
WBF = 8	5/43	11.6
WBF = 10	3/43	7.0
Frequency missing = 1		

Analgesic usage	Frequency	Percent
No	70/118	59.3
Yes	48/118	40.7
Frequency missing = 2		

Analgesic usage without pain (WBF = 0)	Frequency	Percent
No	60/72	83.3
Yes	12/72	16.7
Frequency missing = 2		

No analgesic usage with WBF ≥ 2	Frequency	Percent
WBF = 2	6/17	35.3
WBF = 4	3/9	33.3
WBF ≥ 6	1/17	5.9
Total	10/43	23.3
Frequency missing = 1		

Second analgesic usage with PEP WBF < 6	Frequency	Percent
No	23/26	88.5
Yes	3/26	11.5
Frequency missing = 0		

Second analgesic usage with PEP WBF ≥ 6	Frequency	Percent
No	13/17	76.5
Yes	4/17	23.5
Frequency missing = 0		

Post‐extraction biting injury (PEBI)	Frequency	Percent
No	114/120	95.0
Yes	6/120	5.0
Frequency missing = 0		

Post‐extraction bleeding (PEB)	Frequency	Percent
No	102/120	85.0
Yes	18/120	15.0
Frequency missing = 0		

In the present study, post‐extraction biting injury (PEBI) was rare: only six of the 120 children (5%) reported this discomfort (Table 1) and this discomfort was painful for five of these patients (data not shown). Finally, 15% of children reported PEB (Table 1). For all these children, primary blood control by application of a damp gauze pack directly over the socket was sufficient to stop the bleeding.

### 3.2 PEP, PEBI, and PEB regarding patient's profile or procedure type

Concerning the secondary objectives of this study, determination of possible association between the type of patient or the nature of the intervention and report of discomfort, data from the logistic regression analyses indicated that age, sexes, cooperation, previous experience of extraction, oral hygiene, anxiety (measured by asking the patient if he/she was nervous and based on the activity of the face and legs), accompanying person, as well as tooth position, degree of difficulty (length and number of roots), tooth history, pre‐existing pain or dento‐alveolar abscess (antibiotic before extraction), and number of teeth extracted during a single appointment (one tooth or more than one tooth) were not significantly associated with report of discomfort (Tables 2 and 3).

**TABLE 2:** Factors associated to PEP based upon the patients (age, sexes, socioeconomic levels, cooperation of the child, influence of the accompanying person, number of previous extractions, dental hygiene, anxiety and tears before and during anesthesia or extraction)

Variable	Criteria	Frequency missing	PEP/total patients (percentages)	OR [CI 95%]	*p* Value	
Age	<6 yrs 6–9 yrs >9 yrs	0	6/18 (33.3%) 32/73 (43.8%) 6/27 (22.2%)	0.64 [0.22; 1.89] 1 (ref.) 0.37 [0.13; 1.01]	.42 — .05**	.14
Sexes	Girls Boys	0	19/51 (37.3%) **25/67 (37.3%)**	0.99 [0.47; 2.12] 1 (ref.)	.99 —	
Socioeconomic levels	Favorable Unfavorable	1	19/66 (28.8%) 25/51 (49.0%)	1 (ref.) 2.38 [1.10; 5.11]	— .03***	
Cooperation	Not at all A little A lot off	0	0/8 (0.0%) 10/25 (40.0%) 34/85 (40.0%)	<0.001 [<0.001; >999.99] 1.14 [0.46; 2.81] 1 (ref.)	.99 .78 —	.96
Accompanying person	Absent/neutral Helping Anxiously	1	21/53 (39.6%) 20/56 (35.7%) 2/8 (25.0%)	1 (ref.) 0.85 [0.39; 1.84] 0.51 [0.09; 2.76]	— .67 .43	.71
Number of previous extractions	1 >1	2	23/60 (38.3%) 20/56 (35.7%)	1 (ref.) 0.89 [0.42; 1.90]	— .77	
Oral hygiene	Defective Medium Correct	2	10/24 (41.7%) 21/59 (35.6%) 11/33 (33.3%)	1.29 [0.49; 3.41] 1 (ref.) 0.91 [0.37; 2.22]	.60 — 0.83	.80
Anxiety before extraction	No anxious Anxious	0	28/63 (44.4%) 16/55 (29.1%)	1 (ref.) 0.51 [0.24; 1.10]	— .09	
Anxiety during extraction	No anxious Anxious	6	17/46 (38.0%) 25/66 (37.9%)	1 (ref.) 1.04 [0.48; 2.27]	— .92	
Tears before anesthesia	No Yes	2	39/106 (36.8%) 4/10 (40.0%)	1 (ref.) 1.15 [0.30; 4.31]	— .84	
Tears during anesthesia	No Yes	2	36/88 (40.9%) 7/28 (25.0%)	1 (ref.) 0.48 [0.19; 1.25]	— .13*	
Tears during extraction	No Yes	2	32/87 (36.8%) 11/29 (37.9%)	1 (ref.) 1.05 [0.44; 2.50]	— .91	

*Note*: Percentages were calculated by dividing the number of patients with PEP by the number of patients in each subgroup of criteria for each variable. OR, odds ratio and CI, confidence interval (PEP: 2 missing data).

*p Value <.20; **p value <.10; ***p value <.05.

**TABLE 3:** Factors associated to PEP based upon the tooth and the surgery's characteristics

Variable	Criteria	Frequency missing	PEP/total patients (percentages)	OR [CI 95%]	*p* Value	
Tooth	Mandibular molar Mandibular canine or incisor Maxillary molar Maxillary canine or incisor	1	18/51 (35.3%) 4/7 (57.1%) 19/39 (48.7%) 3/20 (15.0%)	1 (ref.) 2.44 [0.49; 12.15] 1.74 [0.74; 4.08] 0.32 [0.08; 1.25]	— .28 .20 .10*	0.07
Tooth position	Posterior Anterior	0	37/98 (37.8%) 7/20 (35.0%)	1 (ref.) 0.50 [0.19; 1.31]	— .16*	
Existing root length	>2/3 <1/3	1	24/57 (42.1%) 20/60 (33.3%)	1.46 [0.69; 3.08] 1 (ref.)	.33 —	
Indication of extraction	Tooth decay Orthodontic treatment Obstacle Infection Traumatism	1	20/56 (35.7%) 4/16 (25.0%) 7/16 (43.8%) 12/24 (50.0%) 1/5 (20.0%)	1 (ref.) 0.60 [0.17; 2.11] 1.40 [0.45; 4.33] 1.80 [0.68; 4.74] 0.45 [0.05; 4.31]	— .43 .56 .23 .49	.471
Antibiotic before extraction	No Yes	0	31/89 (34.8%) 13/29 (44.8%)	1 (ref.) 1.52 [0.65; 3.56]	— .34	
Pre‐operative pain	No Yes	0	28/89 (31.5%) **16/29 (55.2%)**	1 (ref.) 2.68 [1.14; 6.32]	— .02***	
Dose of local anesthetic	Less than one carpule One carpule More than one carpule	1	12/28 (42.9%) 26/68 (38.2%) 6/21 (28.6%)	1.21 [0.49; 2.96] 1 (ref.) 0.65 [0.22; 1.88]	.67 — .42	.589
Pre‐existing pain	FPS‐R = 0 FPS‐R = 2 FPS‐R = 4 FPS‐R ≥ 6	4	26/77 (33.8%) 10/22 (45.5%) 4/9 (44.4%) 2/6 (33.3%)	1 (ref.) 1.64 [0.62; 4.28] 1.57 [0.39; 6.35] 0.98 [0.17; 5.7]	— .32 .53 .98	.739
Number of teeth extracted	1 >1	0	37/98 (37.8%) 7/20 (35.0%)	1 (ref.) 0.89 [0.33; 2.43]	— .82	

*Note*: Percentages were calculated by dividing the number of patients with PEP by the number of patients in each subgroup of criteria for each variable. OR; odds ratio and CI; confidence interval (PEP: 2 missing data).

The bold values are the statistically significant data.

*p Value <.20; ***p value <.05.

Interestingly, two factors appeared to influence PEP. In the group of patients with PEP, we found a higher percentage of children with unfavorable socioeconomic status compared to children with favorable socioeconomic levels (the odds ratio was 2.38; [CI 95%] 1.10–5.11, *p* = .03). In addition, children with pre‐operative pain, as assessed by the student who performed the extraction using the FPS‐R scale, were significantly more likely to have PEP than those without pre‐operative pain (the odds ratio was 2.68; [CI 95%] 1.14–6.32, *p* = .02; Tables 2 and 3).

Concerning the univariate logistic regression analysis for prediction of analgesic usage, we found a significant difference (*p* < .05) with higher usage of paracetamol in the group with pre‐operative pain and when the tooth extracted had 2/3 of root length. In addition, a significantly higher percentage of parents from the socioeconomically disadvantaged group administered analgesics compared to parents with a favorable socioeconomic level (Table 4).

**TABLE 4:** Univariate logistic regression analysis for (A) prediction of analgesic usage and (A) post‐extraction bleeding (PEB)

A. Variable criteria	Frequency missing	Analgesic usage/total patients (percentages)	OR [CI 95%]	*p* Value
Pre‐operative pain	0			
No		30/89 (33.7%)	1 (ref.)	—
Yes		18/29 (62.1%)	3.22 [1.35; 7.68]	.01***
Existing root length	1			
>2/3		29/57 (50.9%)	2.24 [1.05; 4.74]	.04***
<1/3		19/60 (31.7%)	1 (ref.)	—
Socioeconomic levels	1			
Favorable		20/66 (30.3%)	1 (ref.)	—
Unfavorable		27/51 (52.9%)	2.43 [1.14; 5.18]	.02***
Anxiety during extraction	6			
No		15/46 (32.6%)	1 (réf.)	—
Yes		31/66 (47.0%)	1.83 [0.84; 4.01]	.13**

B. Criteria	Missing	Post‐extraction bleeding	OR [CI 95%]	*p* Value
Pre‐operative pain	0			
No		10/89 (11.2%)	1 (ref.)	—
Yes		8/29 (27.6%)	2.91 [1.03; 8.25]	.045***
Anxiety during extraction	6			
No		3/46 (6.5%)	1 (ref.)	—
Yes		13/66 (19.1%)	3.39 [0.91; 12.64]	.069**

*Note*: Percentages were calculated by dividing the number of patients with analgesic usage or with PEB by the number of patients in each subgroup of criteria for each variable. OR, odds ratio and CI, confidence interval.

The bold values are the statistically significant data.

*p Value <.20; **p value <.10; ***p value <.05.

There were no differences in PEBI between all criteria observed in this study. Unlike PEBI, one criterion was predictive of PEB: children who experienced pain during extraction reported significantly more PEB compared to children without pre‐operative pain (*p* < .05; Table 4).

## 4 DISCUSSION

The incidence of discomfort after deciduous tooth removal needs more attention. In our study, we showed that 37.3% of the children reported pain and that two‐thirds of them expressed mild or moderate pain (WBF ≤ 4). Our data are in the same range of the occurrences of PEP previously reported: 38% (Acs et al., 1986, 1988) and 42.8% (Ashkenazi et al., 2007). Nevertheless, in these studies, the type of teeth extracted (deciduous or permanent) were not discriminated, and scale was not used to assess the presence and the intensity of pain. Compared to adults, children have fewer life experiences, and their reaction toward noxious stimuli should be more objectively based (Acs & Drazner, 1992; Zielinski, Morawska‐Kochman, & Zatonski, 2020). Pain in young children is often difficult to recognize, and their limited communication abilities increase the risk that after deciduous tooth extraction their pain will remain unrecognized or underestimated (Versloot, Hall‐Scullin, Veerkamp, & Freeman, 2008). The most widely used and best validated faces pain scales are the FPS‐R and the WBF for self‐reported measurement of pain intensity in children (Tomlinson et al., 2010; Zielinski et al., 2020). In our study, we have chosen the WBF to evaluate post‐operative pain since children preferred this scale to others and because WBF is suitable for the age range of 3–18 years (Rathi et al., 2019; Tomlinson et al., 2010). Moreover, we decided that the WBF score superior or equal to two, defined pain clinically. This score was used as a cut‐off for the pain because paracetamol is effective for mild to moderate pain, and we asked parents to administer this analgesic as soon as mild pain appeared in order to prevent inadequate pain management and its physical and psychological consequences (AAPD, 2018). Pain appeared during the 3 hr after extraction for 69% of children. This period corresponds to the time in which anesthesia wears off (Odabas, Cinar, Deveci, & Alacam, 2012).

In our study, 23.3% of children reporting PEP did not receive drugs to relieve their pain. Fortunately, the intensity of the PEP experienced by patients was correlated to the use of paracetamol. In fact, 94.1% of children with WBF ≥ 6 took paracetamol. Interestingly, parents did not administer painkillers to one‐third of patients with moderate pain (WBF = 4). These percentages of patients without analgesic administration despite PEP were lower than previously reported (Acs et al., 1986; Acs & Drazner, 1992; Ashkenazi et al., 2007). Possibly, our data pointed out that awareness of PEP relief in children is now an integral part of professional pediatric care. Nevertheless, our study also demonstrated that some parents administered analgesic without pain and, consequently, did not follow the recommendations. The use of a pain medication may depend on parental usage patterns of analgesics and on parental expectations for PEP (Zielinski et al., 2020). Accordingly, we found that presence of roots on the extracted tooth and feeling of pain during extraction, were predictive of analgesic usage. Poor following of the dentist's prescription of analgesic was previously described after dental extractions under general (Jensen, 2012; Wong, Copp, & Haas, 2015). Finally, after primary tooth extraction under local anesthesia, one dose of paracetamol seems to be adequate as we have shown that only seven of 120 patients received a second dose of this analgesic, and a third dose was never required. Berlin and collaborators recently showed through a systematic review that no guidelines can be formulated for the use of oral analgesic administrated after extraction to prevent post‐operative pain in children (Berlin et al., 2019). Our study opens the way to design a new clinical trial to evaluate the efficacy of one dose of paracetamol administered by the pediatric dentist immediately after extraction in order to prevent the appearance of post‐operative pain.

Concerning the second outcome, we find no difference in reported pain prevalence based on the age, sexes, or cooperation of the child before and during anesthesia or extraction, nor did we find an influence of the accompanying person, number of previous extractions, or dental hygiene. Age was previously reported to influence recording and perception of pain, with the older group intellectually much better equipped to self‐report on their sensations post‐treatment (Acs et al., 1986, 1988; Ashley et al., 2012). Nevertheless, in these studies, extractions were probably done on permanent teeth in the older group (10–13 years old), while in our study, none of children underwent extraction of permanent teeth.

Interestingly, socioeconomic status seems to have a significant influence on the pain experienced by the patients and the usage of analgesics. In our study, an unfavorable status of the family was defined by their health insurance category: French medical aid or complementary health care could be associated with low educational levels of the parents, low monthly family income, and a large number of persons in the household. We observed that children living in an unfavorable socioeconomic environment did experience more post‐operative pain and were more likely to receive analgesics. The impact of socioeconomic status on pain was previously described (Dorner et al., 2011; Felipak et al., 2020). Subjects living in less affluent socioeconomic areas reported higher prevalence of pain compared to others. Explanations were that people with lower socioeconomic status have greater chances of suffering from chronic disease, depression, or difficult and painful jobs. However, Dorner and collaborators concluded that there is still a socioeconomic gradient in the report of pain that remains unexplained (Dorner et al., 2011). Moreover, it was shown that living in poorer socioeconomic areas was correlated with frequent use of analgesics after adjustment for age, pain intensity, and physical and mental disabilities (Brekke, Hjortdahl, & Kvien, 2002).

It is well known that effective pain control during primary tooth extraction is critical in pediatric dentistry since painful treatment is shown to be an important etiological factor leading to dental panic (Calis, Cagiran, Efeoglu, Ak, & Koca, 2014). For additional pain relief following dental treatment, that is, restorative and orthodontic treatment and primary teeth extractions under local anesthetic, several studies looked at pre‐operative analgesic usage (Baygin, Tuzuner, Isik, Kusgoz, & Tanriver, 2011; Primosch, Antony, & Courts, 1993; Primosch, Nichols, & Courts, 1995). However, with the data of their meta‐analysis, Ashley concluded that it was difficult to make a firm statement as to the benefit of using pre‐operative analgesics before primary tooth extraction (Ashley et al., 2012, 2016). On the other hand, sedation with midazolam or with nitrous oxide/oxygen are described as facilitating induction of anasthesia and reducing post‐operative behavioral problems after primary tooth extraction (Calis et al., 2014; Galeotti et al., 2016). We find that the prevalence of post‐operative pain was significantly higher in the group of children with painful extractions (pre‐operative pain) than in the group that did not report any pre‐operative pain. Pre‐operative pain could be explained by failure of local anesthesia. As recommended, we aimed to control pre‐operative pain while minimizing the risk of lip and cheek biting by choosing an appropriate local anesthetic at a safe dose (Calis et al., 2014). The local anesthesia used in our study was articaine with adrenaline. This local analgesic agent is considered to be effective for pain management in dental treatment and extraction of primary molars in children and adolescents (Klingberg, Ridell, Brogardh‐Roth, Vall, & Berlin, 2017; Rathi et al., 2019). Compared to lidocaine, children who received articaine during dental treatment reported significantly less pain after the procedure (Bonifacio, 2018).

Finally, the prevalence of post‐operative lip and cheek biting or bleeding was also analyzed. Our data revealed that parents reported only 5% of children with soft tissue injury (PEBI). These results could be explained by the clear advice given to prevent these discomforts. Previous studies found that local anesthetics, especially the inferior alveolar blockers, provoked biting in children (Calis et al., 2014; Hersh, Hermann, Lamp, Johnson, & MacAfee, 1995). In our study, we found no difference associated with tooth position or number of teeth extracted. In addition, the bleeding following teeth extraction could be considered as post‐operative distress, and this negative experience can lead to behavioral disorders and negative attitudes toward future procedures (Coulthard et al., 2006). In spite of recommendations, blood or dislocation of the clot formed in the alveolar space was reported in 18 patients (15% of the children), and the presence of pre‐operative pain was predictive of PEB.

## 5 CONCLUSIONS

This study was a thorough evaluation based upon the patients' characteristics and the tooth and surgery factors that may be used in predicting unpleasant responses by children after deciduous tooth extraction. The dental surgeon, and subsequently the patients, should benefit from advanced knowledge of any variables that may ultimately influence the patients' responses to painful stimuli. The practitioner may consider the pain reported by the young patient during extraction and his/her socioeconomic level in determining the potential for PEP. However, because effective pain control is essential in pediatric dentistry, and in view of the fact that pain after treatment is an etiological factor leading to dental fear, further studies should be performed to produce guidelines and recommendations.

## AUTHOR CONTRIBUTIONS

**Brigitte Alliot‐Licht**: Conceived the idea, designed the study, analyzed the data, drafted the manuscript. **Claire Baillargeau**: Designed the study, collected the data and analyzed the data. **Hugo Charles and Aline Ordureau**: Designed the study, collected the data and analyzed the data. **Sylvie Dajean‐Trutaud, Tony Prud**'**homme, Isabelle Hyon and Assem Soueidan**: Collected the data and critically revised the manuscript. **Emmanuelle Renard**: Conceived the ideas, collected the data and critically revised the manuscript. All authors gave their final approval of the text and agree to be accountable for all aspects of the work.

## Supporting information


**Appendix S1**: Supporting information

**Appendix S2**: Supporting information
