# Supplementary material for: Post‐operative discomforts in children after extraction of primary teeth
Source: Clin Exp Dent Res. 2020 Aug 23;6(6):650–8. doi: 10.1002/cre2.316 (PMC7745079; doi:10.1002/cre2.316)
Supplement: Supplementary file 2 — Appendix S2: Supporting information [file CRE2-6-650-s002.docx]

## Statement of Authorship

The submitting author affirms that all individuals listed as authors agree that they have met the criteria of authorship, agree to the conclusions of the study, and that no individual meeting the criteria of authorship has been omitted. In order to meet the requirements of authorship, each author must have contributed to at least one aspect of each of the four criteria, as listed below. **Please note that for Criteria 1 and 2, authors need only to meet one of the two items listed**. These criteria are not to be used as a means to disqualify colleagues from authorship who otherwise meet authorship criteria by denying them the opportunity to meet criteria 2 or 3. Therefore, all individuals who meet the first criterion should have the opportunity to participate in the drafting, review, and final approval of the manuscript. Any individuals not meeting the criteria may be mentioned in the Acknowledgements section of the manuscript.

Per the criteria defined by the [International Committee for Medical Journal Editors](http://www.icmje.org/recommendations/browse/roles-and-responsibilities/defining-the-role-of-authors-and-contributors.html) (ICJME), please note the contribution made by each author listed in the manuscript. Please select items from the drop down menu.

| Author (Last name, First Initial) | **Criteria 1 (and/or)** | | **Criteria 2 (and/or)** | | **Criteria 3** | **Criteria 4** |
| --- | --- | --- | --- | --- | --- | --- |
|  | substantially contributed to conception or design | contributed to acquisition, analysis, or interpretation of data | drafted the manuscript | critically revised the manuscript for important intellectual content | gave final approval | Agree to be  accountable for all aspects of the work in ensuring that questions relating to the accuracy  or integrity of any part of the work are appropriately investigated and resolved |
| Baillargeau, C | contributed to conception and design | contributed to acquisition, analysis, and interpretation | drafted manuscript | Select item. | gave final approval | agrees to be accountable for all aspects of work ensuring integrity and accuracy |
| Lopez-Cazaux, S | contributed to conception and design | contributed to acquisition | Select item | critically revised manuscript | gave final approval | agrees to be accountable for all aspects of work ensuring integrity and accuracy |
| Charles, H | contributed to conception and design | contributed to analysis and interpretation | Select item | critically revised manuscript | gave final approval | agrees to be accountable for all aspects of work ensuring integrity and accuracy |
| Ordureau, A | contributed to conception and design | contributed to analysis and interpretation | Select item | critically revised manuscript | gave final approval | agrees to be accountable for all aspects of work ensuring integrity and accuracy |
| Dajean-Trutaud, S | contributed to conception and design | contributed to analysis and interpretation | Select item | critically revised manuscript | gave final approval | agrees to be accountable for all aspects of work ensuring integrity and accuracy |
| Prud’homme, T | contributed to conception and design | contributed to analysis and interpretation | Select item | critically revised manuscript | gave final approval | agrees to be accountable for all aspects of work ensuring integrity and accuracy |
| Hyon, I | contributed to conception and design | contributed to analysis and interpretation | Select item | critically revised manuscript | gave final approval | agrees to be accountable for all aspects of work ensuring integrity and accuracy |
| Soueidan, A | contributed to conception and design | contributed to analysis and interpretation | Select item | critically revised manuscript | gave final approval | agrees to be accountable for all aspects of work ensuring integrity and accuracy |
| Alliot-Licht, B | contributed to conception and design | contributed to acquisition, analysis, and interpretation | drafted manuscript | critically revised manuscript | gave final approval | agrees to be accountable for all aspects of work ensuring integrity and accuracy |
| Renard, E | contributed to conception and design | contributed to acquisition, analysis, and interpretation | drafted manuscript | Select item. | gave final approval | agrees to be accountable for all aspects of work ensuring integrity and accuracy |
